# Estimation of Lubrication Layer Thickness and Composition through Reverse Engineering of Interface Rheometry Tests

**DOI:** 10.3390/ma13081799

**Published:** 2020-04-11

**Authors:** Alexis Salinas, Dimitri Feys

**Affiliations:** Department of Civil, Architectural and Environmental Engineering, Missouri University of Science and Technology, Rolla, MO 65409, USA

**Keywords:** lubrication layer, interface rheometer, rheology, concrete, mortar, viscosity, yield stress, pumping

## Abstract

During concrete pumping, a lubrication layer is formed near the pipe wall. Extensive research has been performed on measuring and modeling the properties of this layer and using these values to predict pumping pressures. However, there are numerous discussions in the literature about the composition and thickness of this layer: can it be considered mortar, a micromortar, or is it cement paste? In this paper, possible solutions for the thickness and composition of the lubrication layer are derived from interface rheometry tests. It is assumed that the lubrication layer is composed of one or more concentric layers of paste or micromortar. To accomplish this determination, the rheological properties of the composing paste, mortars with different maximum particle sizes and concrete need to be known. Challenges arising from using different rheometers and from the sensitivity of the paste rheology to shearing are addressed in this contribution. The results show that, mathematically, a single layer of homogeneous paste or mortar with different maximum particle sizes can be responsible for the formation of the lubrication layer. Physically, however, the composing material should contain sand particles to some extent, as particle migration is proportional to the size squared. If the literature results from pumping are applicable to the results obtained in this paper, it seems that the lubrication layer is composed of a mortar with a maximum particle size of around 1 to 2 mm.

## 1. Introduction

It is well known from literature that pumping of concrete requires the formation of a lubrication layer [1]. Absence of this layer would lead to excessive pumping pressures and potentially blocking of the pumpline [2]. Many studies have been performed to predict pumping pressures of concrete, whether through the use of interface rheometers [1,3,4,5,6,7], the Sliper [8,9,10,11], or by measuring the rheology of concrete and its constituent (micro-) mortar [12,13,14]. In the latter case, the thickness of the lubrication layer needs to be known before applying capillary rheometer principles on pipe flow. In case of the interface rheometers and the Sliper, the thickness of the lubrication layer is not necessary, but can also not be retrieved from the measurements. Instead, the lubrication layer thickness is implicitly included in the interface parameters (viscous constant), or in the relationship between pressure loss and flow rate.

Several experiments have been executed to estimate the thickness of the lubrication layer, as well as its composition, either through analysis of the concrete with different colors hardened during flow through a pipe [15], ultrasonic pulse velocity [12,13], or by tracing the velocity profile in a half-open pipe [14]. However, the several studies do not come to a uniform conclusion, as varying lubrication layer thicknesses (in the range from 1 to nearly 10 mm) are reported [9,12,13,14,15]. However, it should be kept in mind that the lubrication layer should have a continuously varying volume fraction of particles as a function of the distance from the solid wall, dependent on their size [16].

Shear-induced particle migration is the mechanism governing the formation of the lubrication layer, but initial non-homogeneous conditions caused by the geometrical wall effect should be kept in mind. Shear-induced particle migration is described in the literature by means of two theories: the diffusive flux model [17] and the suspension balance model [18]. The diffusive flux model developed by Philips et al. [17] attributes shear-induced particle migration to a non-equilibrium in collision frequencies on both sides of the particles, caused by the difference in shear rate. As a consequence, particles migrate and create a concentration gradient increasing from high to low shear rate. This concentration gradient causes two counterfluxes mitigating the primary flux. The first counterflux is the direct consequence of changes in collision frequencies, as less particles are colliding at the high-shear rate side, and more are colliding at the low shear rate side. The second counterflux is caused by the increased viscosity with increasing particle size. After a certain amount of time, a dynamic equilibrium is achieved, and statistically, the concentration gradient of particles remains constant with time. The rate of migration is dependent on the applied shear rate gradient, and on the particle size squared [17]. This means that upscaling from mortar to concrete scale, assuming an increase in particle size by a factor 4, the migration speed increases by a factor 16. As such, coarse particles migrate much faster than finer particles, which may hinder the migration of finer particles after all [19]. This will be an important aspect to consider in the remainder of this paper. However, it should also be kept in mind that the geometrical wall effect is inducing an initial, non-homogeneous boundary condition. This could accelerate the shear-induced particle migration of all particles, and it could cause migration of smaller sizes which could be neglected in case the material is regarded as homogeneous in its initial conditions. A recent study on pumping of concrete has shown that the lubrication layer thickness appeared not to be affected by the coarse aggregate size, but its rheological properties were affected [20].

In light of the discrepancies in reported values for thickness and composition of the lubrication layer, this paper makes an attempt to reproduce the lubrication layer and composition through reverse engineering of the interface rheometry test. It sheds light on the different combinations of lubrication layer thickness and composition, mathematically possible, and the results are compared to existing literature. The reverse engineering is based on the rheological properties of different sub-components of the concrete: the paste, mortars with different particle sizes and concrete itself. However, some significant challenges were encountered using this technique due to the shear-sensitivity of the material, which will be discussed in this paper as well.

## 2. Theoretical Concept

The reverse engineering concept is based on well-known rheological equations for concentric cylinders rheometers: the Reiner–Riwlin equation [21,22]. The volume the concrete takes in the interface rheometer is divided into different artificial concentric rings with different thicknesses. The first layer, closest to the inner cylinder, is composed of paste, and each subsequent layer has an increase in maximum particle. The layers considered have a maximum particle sizes of 300 μm (#50 sieve), 600 μm (#30 sieve), 1.18 mm (#16 sieve), 2.36 mm (#8 sieve), 4.75 mm (#4 sieve), followed by bulk concrete. Knowing the rheological properties of each layer, which will be discussed further, allows calculating the contribution to rotational velocity for each layer, dependent on the applied torque. However, the thicknesses of each layer are unknown, they are in fact the variables, and these will be determined by fitting the thicknesses to approach the torque-rotational velocity measurements in the interface rheometer as well as possible. The contribution of each layer to the rotational velocity can be estimated by modifying the Reiner–Riwlin equation and is given in the following equation. The derivation of this Equation (1) can be found in [23]:

(1) $$\Delta N_{x}=\frac{T}{8\pi^{2}h\mu_{p,x}}\left(\frac{1}{R_{i,x}^{2}}-\frac{1}{R_{o,x}^{2}}\right)-\frac{\tau_{0,x}}{2\pi \mu_{p,x}}ln\left(\frac{R_{o,x}}{R_{i,x}}\right)$$

where: Δ*N_x_* = the contribution of each layer to the rotational velocity (rps)

*T* = applied torque (Nm)

*μ*_*p*,*x*_ = plastic viscosity of sublayer *x* (Pa s)

*τ*_0,*x*_ = yield stress of sublayer *x* (Pa)

*h* = height of inner cylinder submerged in concrete (m)

*R*_*i*,*x*_ = inner radius of layer *x* (m)

*R*_*o*,*x*_ = outer radius of layer *x* (m)

The following constraint should be taken into consideration: *R*_*i*,*x*+1_ = *R*_*o*,*x*_, indicating that layers cannot overlap. Also, the plug radius for each layer should be calculated. If *R*_*p*,*x*_ > *R*_*o*,*x*_, the above equation can be applied. If *R*_*p*,*x*_ < *R*_*i*,*x*_, the layer is not sheared at all, and Δ*N_x_* = 0 for that layer. In case *R*_*i*,*x*_ < *R*_*p*,*x*_ < *R*_*o*,*x*_, the layer is only partially sheared and *R*_*o*,*x*_ should be replaced by *R*_*p*,*x*_ in the equation. The total rotational velocity is the sum of all Δ*N_x_*. One assumption which is made in this process is that the layer thicknesses do not change with varying rotational velocities, which may not be entirely justified.

## 3. Encountered Challenges

Some major challenges were encountered in determining the rheological properties of each layer and comparing them in the general calculations. The first challenge was the difference in output of the rheometers, similar to what was observed in different rheometer comparison campaigns [24,25]. Four rheometers were used, as will be described further, for the paste, mortar, concrete, and interface measurements. A transformation procedure was determined based on a comparison procedure of the used rheometers.

The second challenge was the shear-dependency of the rheological properties of the material [26]. It should be considered that the shear rate inside the paste in mortar or concrete will vary with paste volume, even if a consistent mixing procedure on mortar and concrete is applied. In fact, to be more correct, it will depend on the excess paste layer thickness on the aggregates, and as a consequence, it will depend on the ratio of aggregate volume fraction to maximum packing density [27,28,29]. This means that standard mixing procedures on mortars or concretes will change the rheological properties of the paste with changes in volume fraction relative to maximum packing density, and make it more complicated to compare the data. A separate procedure was developed to estimate the rheological properties of the mortar ensuring the mixing and measurement procedure does not influence the maximum shear rate applied on the paste.

## 4. Materials

### 4.1. Constituent Materials

A commercially available Type I/II (according to ASTM C150 [30]) ordinary Portland cement was employed in this paper. For one of the concrete mixtures, a locally available Class C fly ash was also incorporated. The densities of the cement and fly ash were 3160 and 2930 kg/m^3^, respectively.

**Figure 1:**
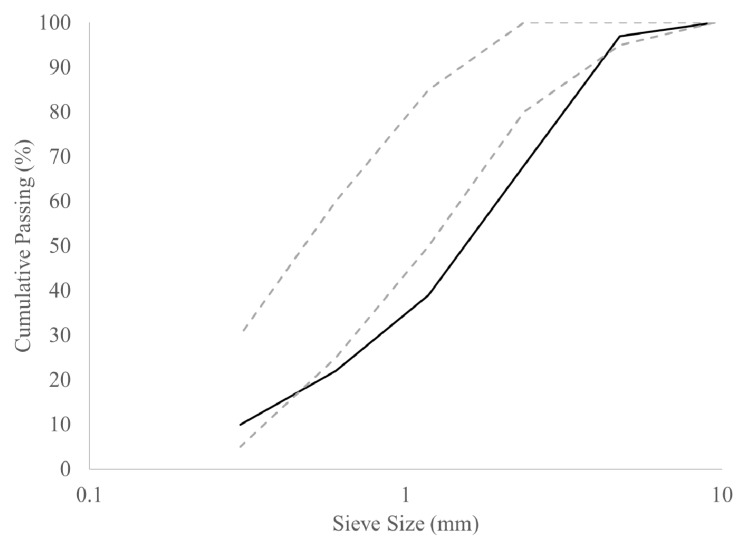
Grain size distribution of crushed sand (full line), accompanied by the ASTM C33 gradation limits (dashed lines).

A locally available crushed sand was selected as its grain size distribution was coarser than a standard natural sand. The coarser sand was preferred to ensure sufficient differences between the volume fractions at different particle sizes. Figure 1 shows the grain size distribution of the sand, accompanied by the gradation limits from ASTM C33 [31]. Table 1 shows the oven dry and SSD densities and the absorption values of different sieved fractions of the crushed sand. For each of the values listed in Table 1, the sieve size indicates the maximum particle size.

The coarse aggregate was a crushed limestone with a nominal maximum aggregate size of 9.5 mm, a density of 2700 kg/m^3^ and an absorption of 0.66%.

The selected admixtures were a PCE-based dispersing admixture and a hydration stabilizer to reduce the effect of hydration on rheological properties. No viscosity-modifying agents or air-entrainers were employed.

For the comparison of the rheometers, a replicate of the National Institute for Standards and Technology (NIST) reference material was produced, using corn syrup, water, and limestone filler [32].

### 4.2. Mix Designs

The work in this paper is divided into three stages: the comparison of the rheometers, the determination of Krieger-Dougherty style curves for yield stress and viscosity of the mortar mixtures, and the estimation of the lubrication layer thickness and composition of the concrete mixtures in the interface rheometer. The following sections contain the information on the mix designs for each of those stages.

#### 4.2.1. Comparing Rheometers

Table 2 represents the mix design employed for the NIST reference material replicate [32].

#### 4.2.2. Krieger-Dougherty Curves for Mortars

The production of the mortars for the Krieger-Dougherty curves followed a procedure which minimizes the impact of mixing of the mortar on the applied shear rate in the paste. The composition of the paste can be found in Table 3. The paste was produced in 9 L batches in an intensive shear paste mixer. Predetermined sand quantities, combined with water quantities to accommodate the water absorption of the sand, were hand mixed with the pastes to obtain volume fractions between 20% and 50% of aggregates. Table 4 gives an example of the sand added to predetermined paste quantities to obtain the correct volume fractions. Table 5 shows the different volume fractions of sand evaluated for each sand size. Higher volume fractions could not be achieved at the finer sand fractions, as these mixtures reached a frictional state in the rheometer [33,34].

#### 4.2.3. Determination of Lubrication Layer Properties in Interface Rheometers

The concrete mix designs for the interface rheometer tests were produced in similar fashion as the mortar mixtures. The paste was created in the intensive shear paste mixer and was added to pre-homogenized aggregates (sand + coarse aggregates) in a 150 L capacity concrete drum mixer. The paste and aggregates were slowly agitated in the drum mixer until a homogeneous mixture was achieved. The slow agitation is expected to keep the shear rate in the paste inside the concrete below the shear rate the paste underwent in the paste mixer. The concrete mix designs contained 38% of paste, 34.7% of the crushed sand and 27.3% of the coarse aggregate by volume. The paste was composed of cement and water for mix designs 1 and 2, at *w*/*c* = 0.35 and 0.40, respectively. Mix design 3 was identical to mix design 2, apart from a replacement of 20% of the cement volume by fly ash. Admixture dosages were kept at 2 and 4 g/kg cementitious materials for the dispersant and the hydration stabilizer, respectively.

## 5. Comparing Rheometers

### 5.1. Rheometers and Procedures

The employed rheometers were the Anton Paar MCR 302 (Graz, Austria) with a serrated concentric cylinders geometry, suitable for cement paste, the ConTec Viscometer (ICI Rheocenter, Reykjavik, Iceland) 6 for mortars with a maximum particle size of 2 mm, two geometries in the ConTec Viscometer 5 for mortar (5S) and concrete (5W) and the ICAR rheometer (Germann Instruments, Copenhagen, Denmark). Table 6 shows the dimensions of each rheometer. For all rheometers, except for the Anton Paar, the measurement procedures were fixed. For the ConTec Viscometer 6, 5S, 5W, and the ICAR rheometer, a maximum rotational velocity of 0.71, 0.50, 0.40 and 0.50 rps, respectively was imposed. The minimum velocity used was 0.03 rps for the ConTec 6, 0.025 rps of the ConTec 5S and 5W and 0.05 rps for the ICAR. All procedures had seven steps, with a pre-shear period at the maximum rotational velocity of 20 s. All tests were executed at three different temperatures: low (2 °C), room (20 °C), and high (35 °C). The reference material and all portable, non-electronic rheometer components were brought to each temperature prior to testing. Temperature of the sample was measured before and after each test. At each temperature, three measurements were performed.

The data for each rheometer were treated by means of the Reiner–Riwlin procedure, transforming the intercept and slope of the torque-rotational velocity relationship into a yield stress and plastic viscosity, respectively. An iterative procedure was applied to correct for plug flow if necessary. Following the procedure to calculate yield stress from the intercept of the curve with the torque axis, the maximum and minimum stress applied during the tests can be derived from the largest and smallest torque values. Minimum and maximum shear rates are determined from the shear stresses and obtained rheological properties. The Bingham model was applied, assuming linear rheological behavior, although some non-negligible shear-thinning was observed. However, no adequate correction procedure for plug flow for non-linear rheological properties is available, and the researchers elected to correct for plug flow and ignore the non-linearity.

For the Anton Paar, the shear rate range and temperature for each of the other rheometers was imposed, resulting in a direct comparison between each rheometer and the Anton Paar. Direct comparisons between the other rheometers are not feasible due to the differences in shear rate ranges.

### 5.2. Results

**Figure 2:**
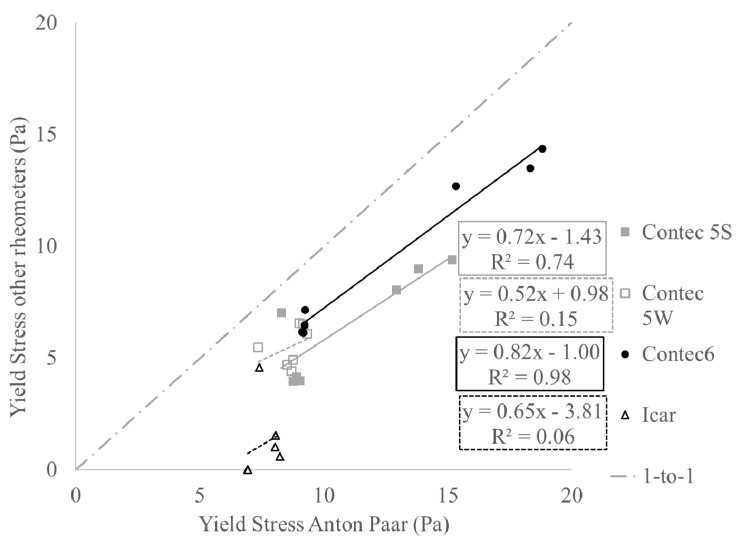
Comparing of yield stress values between all rheometers and the Anton Paar.

**Figure 3:**
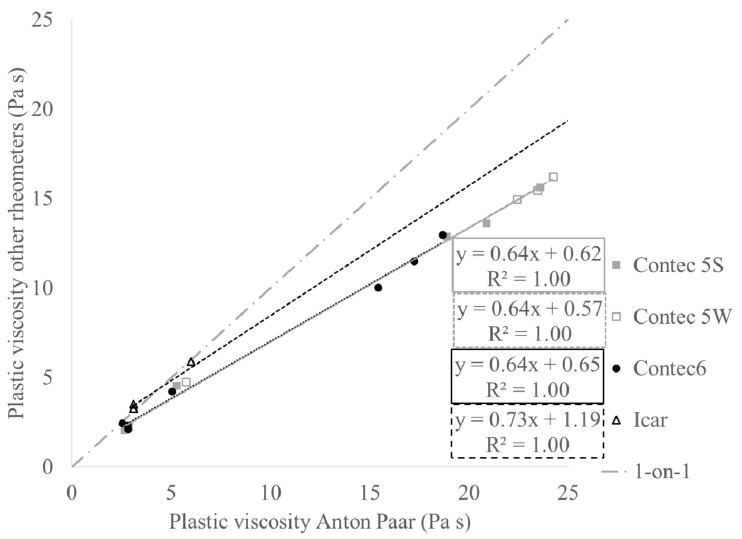
Comparing of the plastic viscosity values between all rheometers and the Anton Paar.

Figure 2 and Figure 3 show the relationships between yield stress and viscosity, respectively, comparing the rheometers with the Anton Paar. For the viscosity, good correlations are observed. For the yield stress, decent correlations are observed as well, but the quality decreases with increasing gap size of the rheometer. For a variety of reasons, beyond the scope of this paper, the correlation between the yield stress of the ICAR and the Anton Paar is of low quality [35]. Instead, it is assumed that the ICAR and ConTec 5W will give the same yield stress values (by lack of other assumptions). By means of the correlations in Figure 2 and Figure 3, transformation equations between the rheometers can be established, through their comparisons with the Anton Paar MCR 302 [35].

## 6. Determination of the Krieger-Dougherty Curves for Mortars

### 6.1. Procedures

All measurements were executed in the ConTec Viscometer 6 following a non-traditional procedure. The pre-shear period, at a rotational velocity of 0.35 rps, was kept intentionally short at 10 s, sufficient to lower the inner cylinder in the sample. The procedure consisted of seven steps of 2 s each, decreasing the rotational velocity from 0.71 to 0.03 rps. The purpose of this adjusted procedure was to minimize the disturbance of the sample when imposing a higher shear rate on the paste in the mortar compared to the paste mixer. Before and after measuring on all mortar samples with different volume fractions of sand, the rheological properties of the paste were determined, and their linear evolution with time served to calculate relative yield stress and relative plastic viscosity, respectively. Apart from the first paste sample, which was returned to the mixer, all other samples were discarded after measuring. Prior to taking the next paste sample to produce a new mortar, it was remixed in the paste mixer.

### 6.2. Results

Based on the ratio of the measured yield stress and viscosity of the mortar samples (considered to be the suspension, indicated with subscript “s” in Equations (2) and (3)), relative to their respective values for paste (considered the medium, with subscript “m”), the relative yield stress (*τ*_0,*r*_) and viscosity (*η_r_*) can be calculated. The Krieger-Dougherty equation for relative viscosity [36], and its counterpart for yield stress [37] are fitted on the data, determining *φ_max_*, *φ_m_*, and [*η*]. *φ_max_* stands for the maximum packing density and *φ_m_* is the percolation threshold: the transition of the material into frictional regime [33,34]. [*η*] is the intrinsic viscosity, taking particle shape, angularity, and roughness into consideration. Separate values for the intrinsic viscosity were fitted for yield stress and viscosity equations.

(2) $$\frac{\eta_{s}}{\eta_{m}}=\eta_{r}={\left(1-\frac{\varphi }{\varphi_{max}}\right)}^{-\left[\eta \right]\varphi_{max}}$$

(3) $$\frac{\tau_{0,s}}{\tau_{0,m}}=\tau_{0,r}=\sqrt{\left(1-\varphi \right){\left(1-\frac{\varphi }{\varphi_{m}}\right)}^{-\left[\eta \right]\varphi_{m}}}$$

**Figure 4:**
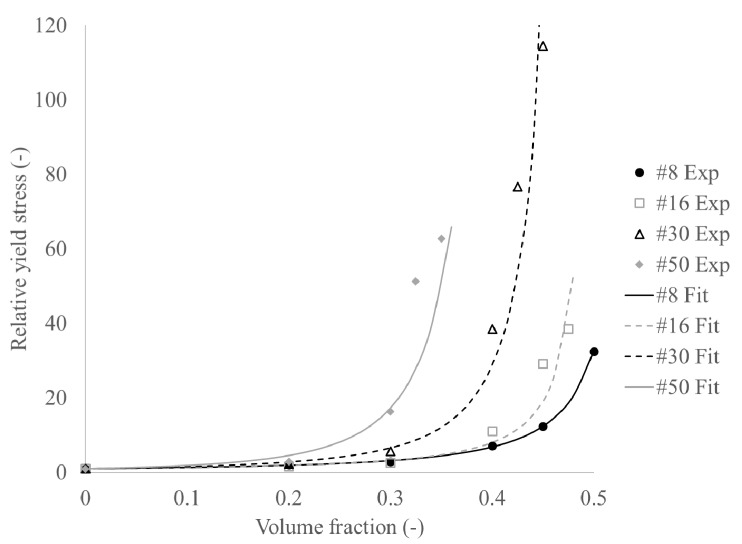
Increase in relative yield stress with increasing volume fraction.

**Figure 5:**
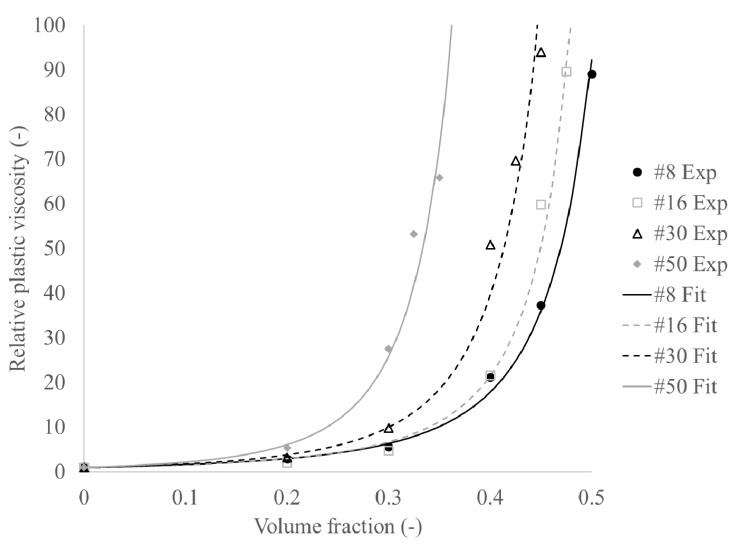
Increase in relative plastic viscosity with increasing volume fraction.

Figure 4 and Figure 5 show the experimental data and curve fits of the data for the yield stress and plastic viscosity, respectively. Table 7 shows the obtained fitting constants. It can be seen that the maximum packing density shows a decreasing trend with decreasing maximum particle size, which is logical seen the decreased polydispersity of the particles. However, the maximum packing densities seem low, which could be partially attributed to the angularity of the crushed sand. However, a second reason could be that the smallest particle size of the sand is of the same order of magnitude as the largest paste particles. As such, a simple distinction between suspending medium and suspended particles is not entirely justified, as the cement particles may interfere with the packing of the smaller sand particles. The percolation threshold values for the yield stress follow a similar trend as the maximum packing density and are systematically between 85% and 90% of the maximum packing density [34]. The intrinsic viscosities appear to increase with decreasing maximum particle size. However, the reason for this behavior is unknown.

Based on the obtained curves, the rheological properties of a mortar with each maximum particle size, at each volume fraction, can now be determined based on the paste rheology. This avoids measuring separately the rheological properties of each mortar layer for the interface rheometer measurements.

## 7. Determination of Composition and Thickness of Lubrication Layer in the Interface Rheometer

### 7.1. Testing Procedure

For this research portion, the paste was mixed separately in the high-shear paste mixer, and the rheological properties were determined with the Anton Paar MCR 302 described above. The procedure consisted of applying a pre-shear for 180 s at 100 s^−1^, following by a decreasing ramp from 100 to 0.1 s^−1^ in 30 s. The concrete rheological properties were evaluated with the ConTec Viscometer 5W, following a similar procedure as for the ConTec Viscometer 6 in order to minimize disturbance of the paste inside the concrete. The interface rheometer consisted of a smooth cylinder of 125 mm in diameter and 200 mm in vertical length [23,38]. The bottom end is conical to facilitate insertion in the concrete, but its contribution to total torque was neglected [36]. The procedure consisted of pre-shearing the concrete for 20 s at 0.5 rps to create the lubrication layer, followed by a stepwise decrease in rotational velocity from 0.5 to 0.05 rps, in seven steps of 5 s each.

The rheological properties of the mortars with different maximum aggregate sizes were estimated based on the volume fraction of sand with that specific maximum aggregate size in the mixture (Table 8), and the Krieger-Dougherty curves established in the section above. For the 4.75 mm (#4) sand particles, as no such curve could be established due to limitations on the particle size in the ConTec Viscometer 6, the curve for the 2.36 mm (#8) particles was assumed.

Once all rheological properties were determined (paste and concrete) or calculated (mortar), they were transformed to correct for the differences in between the rheometers as well, based on the transformation equations obtained with the reference fluid.

### 7.2. Results

Table 9 shows the results for torque values at different rotational velocities in the interface rheometer for each of the measured mixtures. Table 10 shows the measured or estimated rheological properties of each sublayer used for the calculation of the Δ*N_x_* values. In a first strategy, all *R*_*i*,*x*_ values, except *R_i_* itself, were allowed to vary to obtain the best possible solution. This reflects the fact that the lubrication layer is composed of different concentric layers of different mortars. However, the calculated (fitted) thicknesses of these layers were much smaller than their maximum particle size, making this solution physically irrelevant.

**Figure 6:**
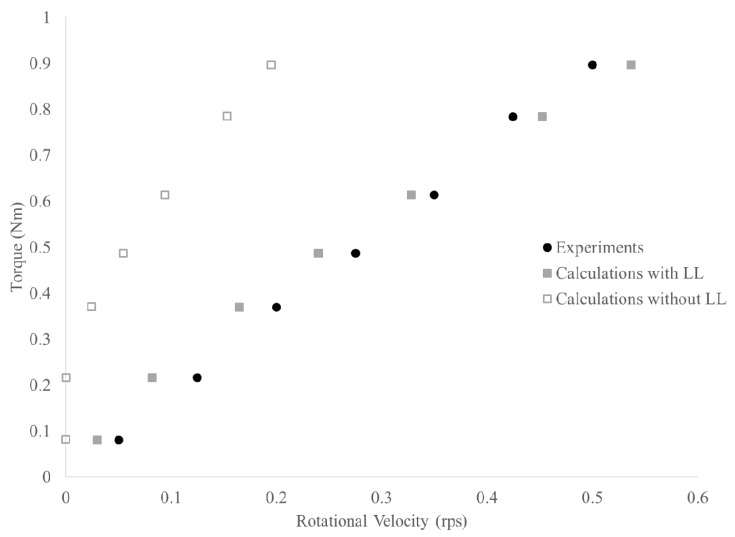
Example of interface rheometer results for mix design 1. The black data is the experiment, the hollow gray points would be the result if the concrete remained homogeneous, while the filled gray points show the best fit with a single mortar layer.

Instead, a second solution strategy was chosen to only vary the thickness of one layer, while keeping all other layers non-existent. In this case, the material in the lubrication layer is assumed to be homogeneous, even if this would deviate from reality. This results in somewhat more adequate layer thicknesses (although still small compared to the maximum particle size). Table 11 shows the obtained fitted thicknesses for each layer separately. It should be kept in mind though that the listed thickness is for that layer specifically, in absence of any other layer. For example, this means that the lubrication layer for mix 1 is composed of a 96 μm layer of paste or a 282 μm layer of mortar with 600 μm particles, or a 1.53 mm thick layer of 2.36 mm particles. Mathematically, for each of the mix designs, the solutions for solely paste or mortar layers up to 1.18 mm particle size provide nearly identical fits on the resulting T-N curve (Figure 6 shows an example). Increasing the particle size of the lubrication layer to 2.36 or 4.75 mm causes more deviation of the points, especially at the lower rotational velocities.

However, the mathematical fits should not determine how the data is interpreted. The results above show that a 2 to 10 mm thick mortar layer (up to 4.75 mm in size) delivers equivalent results as a 0.5 to 2 mm thick mortar layer with 2.36 mm particles as well as a 30 to 125 μm thick cement paste layer. All results are nearly equivalent as the rheological properties of the layers are proportional to each other, especially for the mortar layers with smaller particles. Based on the diffusive flux model, the layer with the largest particles seems the most logical solution, as larger particles migrate faster [16,17]. What is however unknown is how much does the migration of the larger particles hinder the smaller particles to move, etc. [19]. Furthermore, an 8 mm thick layer in an approximately 80 mm gap seems too thick, as that would require an increase in particle volume fraction in the bulk concrete, altering the rheological properties. This is not considered in this analysis. Furthermore, based on these theoretical calculations, the bulk concrete is at least partially sheared in most of the cases, effectively activating the counterflux of particles due to changes in collision frequencies and limiting the migration potential of the particles. On the other hand, stating that the lubrication layer is solely composed of cement paste would be a step too far, as that would require extremely fast migration of all particles in the mixture, especially within the duration of the test (1 min). However, the initial conditions of the sample: i.e., the geometrical wall effect, are not considered when estimating shear-induced particle migration. As such, all of the above solutions might still have validity. However, most results in the literature on effective measurements of thicknesses point toward a 1–2 mm thick layer [5,12,14]. This means that the layer would, most likely, be composed of a mortar with 1 or 2 mm maximum particle size.

## 8. Conclusions

An attempt was made to reverse engineer the composition and thickness of the lubrication layer formed during the execution of an interface rheometry test. Assuming the lubrication layer is formed of *n* concentric layers with different maximum particle sizes, a mathematical model can be established to find the relationship between torque and rotational velocity. By measuring or calculating the rheological properties of cement paste, mortar fractions with different maximum aggregate sizes and concrete, one can calculate the rotational velocity change in a specific layer for each imposed torque. The unknowns in the equation are the thicknesses of each layer, which can be mathematically fitted based on the experimental torque-rotational velocity data.

However, prior to executing such program, two major challenges need to be considered: the differences in the rheometers need to be assessed and the shear rate dependency of the paste inside the mortar and concrete needs to be minimized for the successful execution of the experiments. The different rheometers used in this work to measure the properties of cement paste, mortars and concrete were compared by means of a replicate of the NIST reference material for paste. The behavior of this material was measured in each rheometer and compared to the results in the Anton Paar MCR 302 at the same temperature and for the same shear rate range. Direct correlations for yield stress and viscosity could be established with this latter rheometer, allowing for a 2-step procedure to compare the data.

To eliminate the effect of shearing the paste before and during measurements, the mortar and concrete mixing procedures were adjusted, as well as the rheological measuring procedures. Pre-shear periods and durations of time-steps were minimized. This allowed for the establishment of Krieger-Dougherty style curves for yield stress and viscosity of mortars with varying maximum particle sizes. With each decrease in maximum particle size, a decrease in calculated maximum packing density, a decrease in percolation threshold for friction and an increase in intrinsic viscosity was noted. These curves were used to calculate the rheological properties of different mortar layers in the interface rheometer test, based on the measured paste rheology.

For the interface rheometry tests, the thicknesses of each layer were mathematically determined to fit the experimental torque-rotational velocity data. However, this led to layer thicknesses much smaller than the maximum particle, which does not make sense physically. Instead, the required thickness of a single layer was determined to match the torque-rotational velocity data, for each of the layers considered. Each time, a mathematically adequate solution was obtained, regardless of the considered layer. However, physically, the largest particles migrate faster, resulting in more plausibility for the solution with the largest particles. On the other hand, excessive layer thicknesses might not be fully representative of the reality due to increased counterfluxes limiting shear-induced particle migration. Similarly, the initial condition: i.e., the geometrical wall effect, is not considered in the proposed solution. A majority of the results in the literature report a lubrication layer thickness of approximately 1 to 2 mm. If the results in this paper can be applied to pumping conditions, despite the difference in applied shear rate, the many sources of errors in measurements and estimates, etc., this would mean that the maximum particle size in the lubrication layer would be in the order of 1 to 2 mm.

## Figures and Tables

**Table 1:** SSD, oven dry densities and absorption values of the crushed sand, for each of the sieved fractions, indicated by their maximum particle size.

Sieve Size #	SSD Density	OD Density	Absorption
8 (2.36 MM)	2580 kg/m^3^	2550 kg/m^3^	1.27%
16 (1.18 MM)	2560 kg/m^3^	2520 kg/m^3^	1.50%
30 (0.60 MM)	2560 kg/m^3^	2520 kg/m^3^	1.77%
50 (0.30 MM)	2550 kg/m^3^	2490 kg/m^3^	2.42%

**Table 2:** Mix design of NIST reference material replicate.

Material	Quantity (KG)
Corn Syrup	18.63
Distilled Water	5.89
Limestone Powder	42.68

**Table 3:** Composition of 9 L of paste for the determination of the Krieger-Dougherty curves of the mortar mixtures.

Material	Quantity
Cement	9.97 kg
Fly Ash	2.49 kg
Water	4.98 kg
PCE	32.4 g
HS	37.4 g

**Table 4:** Quantities of sand and paste needed to obtain specific volume fractions in mortar samples.

Volume Fraction	Sand	Cement Paste
20%	634 g	1873 g
30%	950 g	1639 g
40%	1267 g	1405 g
45%	1426 g	1288 g
50%	1584 g	1171 g

**Table 5:** Evaluated volume fractions for each sand portion with listed maximum aggregate size.

Sieve Size	20%	30%	35%	40%	42.5%	45%	47.5%	50%
#8	X	X		X		X		X
#16	X	X		X		X	X	
#30	X	X		X	X	X		
#50	X	X	X	X				

**Table 6:** Dimensions, minimum and maximum shear rate of each rheometer.

Rheometer	R_I_ (MM)	R_O_ (MM)	H (MM)	Min Shear Rate (1/S)	Max Shear Rate (1/S)
ANTON PAAR	13.3	14.5	40.0	Variable	
CONTEC 6	50	61.5	Variable	0.4	21.1
CONTEC 5S	65	82	Variable	0.4	13.2
CONTEC 5W	100	145	Variable	0.3	6.8
ICAR	63.5	143	127	0.7	3.9

**Table 7:** Fitted values for Krieger-Dougherty equations for yield stress and viscosity.

	ΦM	[*η*] FOR YS	ΦMAX	[*η*] FOR PV
**#8**	0.565	6.27	0.628	4.53
**#16**	0.511	6.01	0.579	4.52
**#30**	0.501	9.02	0.591	5.51
**#50**	0.455	12.38	0.494	7.05

**Table 8:** Volume fractions for each sand fraction used to estimate the rheological properties of each mortar layer.

Sieve Size	Volume Fraction
#4	0.47
#8	0.38
#16	0.26
#30	0.17
#50	0.09

**Table 9:** Measured torque values in the interface rheometer for each mixture at the pre-determined rotational velocities.

ROT Velocity	Torque MIX 1	Torque MIX 2	Torque MIX 3
0.50	0.90	0.58	1.02
0.42	0.78	0.53	0.89
0.35	0.61	0.46	0.78
0.27	0.49	0.40	0.70
0.20	0.37	0.33	0.54
0.12	0.22	0.25	0.44
0.05	0.08	0.16	0.30

**Table 10:** Measured (paste and concrete) or calculate (mortar) rheological properties of each hypothetical layer in the interface rheometer.

	Paste	#50	#30	#16	#8	#4	Concr
**YS MIX 1**	0.7	1.3	1.6	1.9	4.2	12.4	39.7
**PV MIX 1**	0.13	0.26	0.38	0.63	1.88	6.63	33.5
**YS MIX 2**	0.6	1.1	1.4	1.6	3.6	10.5	54.4
**PV MIX 2**	0.09	0.18	0.27	0.46	1.35	4.78	24.3
**YS MIX 3**	0.5	0.8	1.1	1.2	2.8	8.2	50.6
**PV MIX 3**	0.07	0.14	0.21	0.34	1.02	3.62	26.6

**Table 11:** Required thickness of an individual layer to be considered to be the lubrication layer in the interface test.

MAX AGGR. Size	MIX 1	MIX 2	MIX 3
#200	0.096 mm	0.123 mm	0.033 mm
#50	0.191 mm	0.244 mm	0.066 mm
#30	0.282 mm	0.362 mm	0.098 mm
#16	0.48 mm	0.61 mm	0.17 mm
#8	1.53 mm	1.96 mm	0.51 mm
#4	7.46 mm	9.59 mm	2.08 mm
